# Exploring the Frequency, Intensity, and Duration of Loneliness: A Latent Class Analysis of Data from the BBC Loneliness Experiment

**DOI:** 10.3390/ijerph182212027

**Published:** 2021-11-16

**Authors:** Pamela Qualter, Kimberly Petersen, Manuela Barreto, Christina Victor, Claudia Hammond, Sana-Arub Arshad

**Affiliations:** 1 Manchester Institute of Education, University of Manchester, Manchester M13 9PL, UK; kimberley.petersen@manchester.ac.uk (K.P.); arshad18@live.co.uk (S.-A.A.); 2 Psychology Department, University of Exeter, Exeter EX4 4PY, UK; M.Barreto@exeter.ac.uk; 3 College of Health and Life Sciences, Brunel University, London UB8 3PH, UK; Christina.Victor@brunel.ac.uk; 4 BBC Radio 4, Broadcasting House, Portland Place, London W1A 1AA, UK; claudia.hammond@bbc.co.uk

**Keywords:** loneliness, latent class analysis, online survey, measurement

## Abstract

Almost all measures of loneliness have been developed without discussing how to best conceptualize and assess the severity of loneliness. In the current study, we adapted the four-item UCLA, so that it continued to measure frequency of loneliness, but also assessed intensity and duration, providing a measure of other aspects of loneliness severity. Using data from participants resident in the UK who completed the BBC Loneliness Experiment (N = 36,767; F = 69.6%) and Latent Class Profile Analyses, we identified four groups of people who scored high on loneliness on at least one of the three severity measures. Duration of loneliness often over months or years seemed to be particularly important in distinguishing groups. Further, group membership was predicted by important demographic and psychological variables. We discuss the findings in terms of implications for research and practice. We highlight the need to explore these profiles longitudinally to investigate how membership predicts later mental and physical health, and well-being.

## 1. Introduction

Loneliness is commonly defined as an unpleasant psychological reaction to the absence of desired social relations [1] related to either the quality or the quantity of social connections. It is an unpleasant experience for individuals, accompanied by psychological distress [2]; whether experienced in childhood, adolescence, adulthood, or old age, when describing loneliness, people mention emotions such as sadness, emptiness, stress, frustration, anger, anxiety, and boredom [3]. Loneliness has important implications for physical and mental health, again, regardless of age [4], which means it has become a public health and policy concern. 

The severity of loneliness has been most often quantified as the frequency with which loneliness is experienced. Indeed, the most widely used measures of loneliness, across all age groups, ask about how often loneliness is experienced (never–always), creating a score that ultimately measures how often loneliness—or related feelings or behaviour that accompany it—is experienced [5]. However, the current focus on frequency of the loneliness experience is not the only way to explore the severity of loneliness, which may, instead, be best conceptualized in terms of intensity or duration, or a combined assessment. Indeed, in his writings, Weiss (1973) highlighted that both the frequency and intensity of the loneliness experience should be examined more in research [6], but the latter appears almost absent from academic study. When he discussed frequency, Weiss talked in terms of length of time when loneliness was experienced, which appears to reflect duration, rather than frequency. Despite frequency, intensity, and duration of loneliness being viewed as important aspects of loneliness for some time, there has been no exploration of which rating parameters, or combination, provide the most valid indicators of loneliness severity. Most scales were developed without consideration of how best to conceptualise and assess the severity of loneliness. In the current study, we used data from the BBC Loneliness Experiment to explore how three measures of severity of loneliness-frequency, intensity, and duration-come together to create subtypes of loneliness.

### 1.1. Different Ways to Measure Severity of Loneliness

The UCLA Loneliness Scale, and its various derivatives, is the most common scale of loneliness used by researchers [7]. There are other questionnaires that are also commonly used [5], but all questionnaires contain statements referring to the frequency/persistence of the feelings/behaviour that accompany the loneliness experience (e.g., never-always present). While the statements are discussed in terms of them being about the frequency of loneliness, few loneliness measures include a timeframe for reference, so we actually do not know how often loneliness occurs. In measurements of other emotion-focused experiences, such as depression, the most common timeframes are the past one or two weeks, but in the original UCLA loneliness scale and its derivates, there are no mention of a timeframe. Other loneliness questionnaires for older adolescents and adults suffer from the same problems. If we are measuring the frequency with which loneliness is experienced we would expect to see a temporal reference period with appropriate response options (e.g., daily, 4–5 days per week). We argue that what current measures address is the *persistence* of loneliness-related emotions and behaviours, i.e., are those always present versus often, sometimes, or never present? What is missing are other dimensions of the severity of loneliness, including duration of loneliness (e.g., whether loneliness lasts a day, a week, a month, a year) and intensity of loneliness (e.g., the level of distress the loneliness causes: severe vs. moderate vs. mild). Furthermore, in papers reporting the development of loneliness measures, no information is provided for why a frequency format was chosen to measure loneliness rather than, or in addition to, duration or intensity. To fill the gap in knowledge about the role of different measures of severity of loneliness, the aim of the current study was to explore the relationship between scores for frequency, intensity, and duration of loneliness. 

### 1.2. Using Latent Class Analysis to Explore Subtypes of Loneliness

We were also interested in whether the different severity aspects of the loneliness experience came together to create loneliness subtypes. Using Latent Class Analysis (LCA), we explored whether there were groups of people that showed similar patterns across the different severity aspects of loneliness, and whether we could predict group membership from demographic information and other variables completed as part of the BBC Loneliness Experiment. LCA identifies underlying/hidden/latent subgroups in the population by exploring patterns of similarities and differences between respondents [8]. It does that by analysing individuals’ patterns of behaviour, such as severity of loneliness indicators, and finding common types, called classes [9].

It is important for researchers in the field of loneliness to explore whether there are subgroups of people within the general population who experience similar patterns of loneliness across the severity aspects of frequency, intensity, and duration, so that appropriate intervention can be delivered. The subgroups can be studied further to investigate how prevalent they are, what causes them, what future outcomes they predict, and whether loneliness class changes over time [8], so that a targeted approach to loneliness prevention can be established. In the current study we used LCA to explore the data on frequency, intensity, and duration of loneliness from the BBC Loneliness Experiment. LCA was used to determine whether distinct subgroups of people could be identified based on the different loneliness severity scores, and to see if they could be predicted by important demographic and psychological variables. 

### 1.3. The Current Study

In the current study, using data from participants residing in the UK who completed the BBC Loneliness Experiment, we explored different aspects of severity of loneliness. First, we determined whether there were relationships between scores for frequency, intensity, and duration of loneliness. Second, we examined whether there were subgroups of people based on those data, and explored whether membership of those different subgroups was predicted by demographic and psychological variables. 

## 2. Material and Methods

### 2.1. Participants and Procedure

Participants took part in an online survey launched on BBC Radio 4 and the BBC World Service, and covered by newspapers and broadcast media. Participants who were interested could access the study online. They were first provided with information about the study. Those who agreed to participate answered a range of questions about their social life and their experiences with loneliness. A total of 54,988 people of ages 16–99 years completed the survey. In this paper, we report data related to the experiences of loneliness from the 36,767 participants who resided in the UK, so our sample was as homogenous as possible given the effects of culture on loneliness [10]. Missing data analysis for the loneliness indicator showed that, for 1853 cases, data were missing on all indicator variables, so those cases were excluded from the analysis. That left 34,914 cases in the initial LCA analysis whose experiences of loneliness we could examine (age range 16–99, F = 69.9%). 

Selected variables from the BBC Loneliness Experiment were used as predictor variables in our LCA. Most were chosen given that they had previously predicted frequency of loneliness [11]; others we expected to be important predictors of loneliness based on our previous and current work. Missing data analysis for these variables ranged from 0–23.1% of cases. Where there are missing data on covariates, Mplus deletes cases in a listwise fashion, which meant that 15,306 cases were deleted, leaving 19,608 cases in the covariate analysis. Demographics of participants whose data are used in the current paper are described in Table 1.

### 2.2. Measures

**Loneliness** was measured by asking participants to answer questions from the four-item UCLA Loneliness Scale [12]: Do you feel a lack of companionship? Do you feel left out? Do you feel isolated from others? and Do you feel in tune with people around you? (reversed coded). As in the original version of the measure, each item was rated by participants on frequency (how often does that happen? Never (0)–Very often (5)). In addition, participants were asked to rate each item on intensity (how intense is that feeling? Not intense at all (1)–Very intense (5)) and duration (how long does that feeling last when it occurs? 1 = hours, 2 = days, 3 = weeks, 4 = months, 5 = longer). Each scale was summed across the four items to create total scores for loneliness frequency, intensity, and duration. The measures were reliable (αs for frequency, intensity, and duration = 0.847, 0.862, and 0.879 respectively). 

**Predictors of Severity of Loneliness Classes.** Participants provided demographic and psychological information that were used in the analyses to explore whether they predicted class membership. Participants provided information on their *Age*, which was categorised as follows for the initial analyses: 16–24, 25–34, 35–44, 45–54, 55–64, 65–74, 75+ years). For the LCA analyses, participants were categorised into three groups (16–34, 35–64, 65+ years). Employment/education status was categorised into two groups (employed, retired, or studying [part or full-time] vs. not employed). Participants also indicated the number of *hours they spent alone* (on average, every 24 h), their *marital status* (single, in a relationship but not living together, married or cohabiting, separated or divorced, and widowed, which we recoded for analyses as in a relationship or not (0 = in a relationship; 1 = not in a relationship)), whether they were a *carer* or not (0 = no, 1 = yes), had *dependents* or not (0 = no, 1 = yes), and were an *immigrant* to the UK or not (0 = no, 1 = yes). In addition, participants provided information on sexual orientation, information about life events they had experienced, whether or not loneliness had ever been a positive experience for them (leading to a positive outcome), was controllable and changeable, the extent to which they had experienced everyday discrimination, social capital, relational mobility, and health. *Socio-economic Status*. The MacArthur Subjective Social Status measure (MSSS) [13] depicts individuals at all levels of society within their country using a 10-rung ladder, with the highest rung representing those with the most money, most education, and the most respected jobs, and the lowest rung representing those with the least money, least education, and the least respected jobs. Individuals in the BBC Loneliness Experiment completed the MSSS, being asked to place themselves on the ladder, relative to others in their country. *Sexual Orientation*. We used the Kinsey Sexual Behaviour Scale [14], which ranges from “exclusively heterosexual” to “exclusively homosexual” and adds an option of “asexual”. We split the sample into two groups for analytic purposes (heterosexual/sexual minority). *Number of Significant Life Events in the Last Year.* We used a revised version of the Life Change Index Scale [15] that excluded the item referencing Christmas (to ensure the scale was meaningful irrespective of religious affiliation) and summed the number of life events experienced by each participant in the preceding 12 months. *Positive Experiences of Loneliness*. Participants were asked “Is the experience of loneliness positive for you?” and we dichotomised responses into No = 0, and sometime/yes = 1. Here, our aim was to explore whether participants ever felt that loneliness led to positive outcomes, such as reconnection, social engagement, positive time alone, reflection, among others. *Ability to Change Loneliness*. Participants answered the following question: ‘If you think about when you feel lonely, to what extent do you agree or disagree that the feeling of loneliness is caused by something you can change?’ using a rating scale 1 (strongly disagree) to 5 (strongly agree). *Ability to Control Loneliness*. Participants were asked ‘If you think about when you feel lonely, to what extent do you agree or disagree that the feeling of loneliness is caused by something you can control?’ and answered 1 (strongly disagree) to 5 (strongly agree). Participants’ experiences with *Discrimination* were assessed using the five-item version of the Everyday Discrimination Scale [16]. Participants indicated how often each of the five items had happened to them. An example item is “You are treated with less courtesy or respect than other people” (1 = never to 7 every day; α = 0.79). *Social capital* was measured using a seven-item scale [17] with items including “People around my local neighbourhood are willing to help their neighbours”, which were rated from 1 (strongly disagree) to 5 (strongly agree; α = 0.82). *Relational mobility* was measured using a 12-item scale [18]. Participants were asked to think about the people in their immediate community and to indicate to what extent they agreed with each item. An example item is “They are able to choose, according to their own preferences, the people whom they interact with in their daily life” (1 = strongly disagree to 6 = strongly agree; α = 0.90). *Self-rated health.* Participants were asked to note what their health was like in general, using the rating scale 1 = poor, 2 = fair, 3 = good, 4 = very good, or 5 = excellent. 

### 2.3. Overview of Analysis Plan

In the first step in our analyses, we explored the correlations between scores of frequency, intensity, and duration of loneliness. In the second step, the extent of missing data on indicator and predictor variables that would be included in the LCA analyses was examined. Latent Class Analysis (LCA) was then used to identify groups of individuals with different profiles of loneliness, based on the mean frequency, intensity, and duration of loneliness. 

LCA is a statistical approach that is used to probabilistically assign individuals to subgroups based on their patterns of response to questionnaire items. In the first stage of the current LCA, three continuous indicators of loneliness (mean frequency, intensity, and duration of scores) were used for the latent class analysis. Local independence was assumed, i.e., all correlations between these items are explained through the latent class structure. A series of models, with varying numbers of classes (1–14), were fit to the loneliness class data to determine the optimal number of latent classes that underlie the loneliness severity data. We used several criteria in guiding our decision about the optimal number of latent classes: one set of criteria had to do with the substantive meaning and theoretical conformity of the extracted classes [19], which was determined in part by whether they were predicted by the covariates. We used statistical tests and indices to help in this decision process based on recommendations in the field [9]: The Akaike Information Criteria (AIC), Bayesian information criteria (BIC), Adjusted BIC (ssaBIC), Entropy, and the Lo-Mendell-Rubin likelihood ratio test (LMR-LRT). Lower values on AIC, BIC and ssaBIC indicate better model fit. Significant *p*-values on the LMR-LRT indicate that the k model is a significantly better fit to the data than the k-1 model. 

As suggested in the literature [20,21] fit statistics were used to narrow down the number of models to a smaller number of candidate models. Then, the substantive meaning of classes was taken into account (i.e., to what extent are the classes interpretable in line with current theory and research? Which model identifies classes which are most useful for understanding loneliness?). In addition, parsimony and smallest class size were considered. 

In the second stage of the LCA, we added covariates to the model using the three-step method [22]; MPlus R3STEP command was used [23]. In LCA, including covariates in the latent class model can change the structure of the classes thereby changing their meaning [23]. The three-step model attempts to overcome this issue by preserving the initial latent classes along with measurement error and then adding covariates to the model [22]. In the first step, class enumeration is carried out using the indicators only. In step 2, the most likely class variable and average probability of being in the most likely class (i.e., uncertainty rate/measurement error) are calculated. In the 3rd step, the auxiliary variables are included in the model and logistic regression is carried out between the predictor variables and class membership, with measurement error accounted for. Results indicate the relationship between the auxiliary variables (i.e., the predictor variable) and class membership.

## 3. Results

Table 2 shows the sample means for the different aspects of severity of loneliness. Total Frequency scores for the full sample (M = 10.83) are comparable to scores found in other studies [8], including across age groups. Means suggest that the feelings and behaviour that accompany loneliness did not happen that often, although they were more common among those participants ages 16–24 and 35–44 years than all other age groups (*F* = 63.110, *p* < 0.001). Means for Intensity show that the experience of loneliness was not that intense, although it was significantly more intense for participants ages 16–24 years (*F* = 91.623, *p* < 0.001). Duration scores suggest that overall, participants aged over 65 years experienced loneliness for shorter durations than those in any other age groups (*F* = 45.497, *p* < 0.001). Table 3 shows there are significant and high correlations between the three measures of severity of loneliness, with *rs* > 581 to 0.840, with variation across age groups. 

Table 4 shows the fit statistics for each LCA model. The BIC, AIC, and ssaBIC continued to decrease for each class solution, suggesting a solution with >14 classes fit the data best. However, when information criteria continue to decrease in this way the relative decrease in information criteria may be more useful. Tests of relative fit and LMR-LRT provided further information. LMR-LRT indicated that each k class model was a significantly better fit to the data than the k-1 class model until the 11-class model was reached. LMR-LRT was nonsignificant for the 12-class model, indicating that the more parsimonious 11-class model was preferred. 

Given the information noted above, statistical information narrowed down the models to an 11-class model or a model with >14 classes. The substantive meaning of classes was considered to help us select the best model. The four-class model did not have classes that represented groups of particular interest, such as those that reported different durations of loneliness, whereas the 11-class model identified groups of substantive interest (e.g., those that experienced average frequency and intensity of loneliness, but long duration, and those with high levels of frequency and intensity of loneliness, but only for short durations). Beyond that point, the additional classes were not of substantive interest: the additional classes represented only variations on classes found in preceding models. Therefore, models with more than 11 classes were rejected. Thus, the 11-class model was selected. This model also had good entropy (0.811), indicating that individuals were classified accurately into classes. 

### Latent Class Profile Analysis

Table 5 details the means for the different aspects of severity of loneliness for each class identified in the 11-class model. The 11-class solution includes four lonely groups that showed different profiles of loneliness: (1) average frequency and intensity of loneliness, but long duration (of a magnitude that corresponds to years experiencing the unpleasant psychological impact of loneliness) (group 6); (2) high frequency and intensity, and long duration of loneliness, so the group that experienced the most severe loneliness (group 9); (3) high frequency and intensity of loneliness, but short duration, equating to days or weeks (group 10); and (4) high frequency and intensity of loneliness, and average duration, equating to months (group 11). 

Table 6 (and Table 7 for easy viewing) show that, *compared to the ‘non-lonely’ groups*, each of these ‘lonely’ groups spent more hours alone, experienced more challenging life events in the preceding 12 months, experienced more prejudice, reported worse health, and reported less social capital and relationship mobility. Apart from group 6, the ‘lonely’ groups were also more likely than the ‘non-lonely’ groups to report that they had experienced positive aspects of loneliness and less likely than the ‘non-lonely’ groups to be 65+ years. In addition, ‘lonely’ groups 10 and 11 were less likely than the ‘non-lonely groups’ to see loneliness as controllable. ‘Lonely’ groups 6 and 9 were more likely than ‘non-lonely’ groups to be male, and less likely to be ages 16–24 years; ‘lonely’ group 6 were less likely to be in a relationship than the non-lonely groups. 

Table 6 and Table 7 (Table 7 for easy viewing) also show *differences across the ‘lonely’ groups*. For example, class 6 and 9 (both long durations of loneliness) were characterised by the lowest SES, the worst health, and were more likely to see loneliness as controllable than the other two lonely classes. They were also more likely than the other lonely classes to be male and to be over 65 years of age. Compared to participants in the other lonely classes (9, 10, and 11), participants in class 6 (long duration, but average levels of frequency and intensity) were most likely amongst the ‘lonely groups’ not to be in a relationship, spent the fewest hours alone, reported that they had not experienced loneliness as positive, and experienced the least discrimination of the lonely groups. Participants in class 9 spent the most time alone of the lonely groups, experienced the most discrimination in their daily lives, but reported that they had experienced loneliness as positive. In addition to having higher SES and being less likely to be ages 65+ years than classes 6 and 9, participants in classes 10 and 11 were more likely to be of ages 16–24 years than classes 6 and 9 and spent more time alone than class 6, but less than class 9. Groups 10 and 11 were also more likely than classes 6 and 9 to report loneliness as controllable, with class 10 also being more likely than class 11. Participants in class 10 were less likely to be male than classes 6 and 9, had the best health of all lonely classes, and better relational mobility. Class 11 reported more social capital than the other groups.

## 4. Discussion

Up until now most research on loneliness has measured it in terms of the frequency of negative feelings and behaviour that accompany that experience (e.g., whether those occur never, often, or always). Using such measures, loneliness is associated with a range of outcome measures, including mental and physical health, and well-being [4]. Amongst adults, at least, being 16–24 years of age, single, of poor health, not feeling a sense of belonging to the neighbourhood, and low trust in the local community are consistent risk factors for scoring high on frequency measures of loneliness [11]. However, the frequency with which one experiences loneliness, while important, does not capture other dimensions of the experience, such as how intense it is or how long it lasts. Loneliness that is intense or long lasting, irrespectively of whether it is a frequent experience, is also likely to have important consequences, but those aspects of loneliness are not often captured in existing research. That is, there are other ways to measure severity of loneliness, and in the current study we showed that, whilst correlated, different aspects of loneliness severity (frequency, intensity, and duration) appeared to be important for understanding the diversity of loneliness experiences.

### 4.1. Different Loneliness Experiences

Our analyses identified four groups of people who scored high on loneliness on at least one of the severity measures. The group for which loneliness was most severe (i.e., where scores were high on all three dimensions of loneliness (frequency, intensity, and duration): Class 9) differed from all the other groups by including participants who spent *the most* time alone and reported *the most* discrimination. 

**Average vs. High Frequency and Intensity**. Insights into the differences between people who reported average vs. high loneliness frequency and intensity can be attained by examining what distinguishes Class 6 participants. This was the only group of ‘lonely participants’ who reported average levels of loneliness frequency and intensity, whereas Classes 9, 10, and 11 all included participants who reported high levels of frequent and intense loneliness. Our analyses showed that Class 6 differed from the other three classes of ‘lonely participants’ because it was associated with less time alone, fewer experiences with discrimination, and *less* likelihood of being in a relationship. The latter finding seems surprising, but it is possible that, for these participants, loneliness was experienced within relationships, highlighting the fact that being in a relationship does not fully protect against loneliness. In sum, the difference between participants who reported average vs. high frequency and intensity was how much time they spent alone, how much discrimination they experienced, and whether they were in a relationship.

**Short vs. Long Duration**. In turn, the difference between participants who reported short, medium, and high duration of loneliness can be ascertained by comparing the characteristics of Classes 6 and 9 with those of classes 10 and 11. This comparison reveals that those who report the lowest duration of loneliness (Class 10) are more likely to be male, in better health, and have high relational mobility. This might suggest that men are more likely than women to quickly address loneliness, but that needs to be explored prospectively. There are other reasons that men in good health and relational mobility may experience less lonely, and that may mean they are able to overcome loneliness more quickly: they are more highly privileged, experience less discrimination, and have better access to health care where they do not experience systemic, misogynistic discrimination. The findings could also suggest that loneliness that does not last long is good for one’s health—though it could also be that healthy people are better able to ensure their loneliness is short-lived, even if frequent and intense. The reported higher levels of relational mobility by Class 10, suggest that, comparatively, these individuals are not only afforded an environment to freely form more new relationships as compared to Class 6, 9 and 11, but it also attributes to them certain mindsets and behaviours such as higher self-esteem, closer friendships, and more proactive interpersonal behaviours such as self-disclosure [18]. Because both self-disclosure and having more social support have the potential to protect from loneliness [24], this group may be protected from loneliness of long duration because they are able to find others to confide in. Regarding relational mobility, the most likely scenario is that relational mobility allows people to ensure their loneliness is short-lived, rather than short periods of loneliness enhance relational mobility. Further, participants who reported average duration of loneliness (Class 11) also indicated high social capital; it is not surprising that having a social network ‘out there’ makes it easier to get out of experiences with loneliness. 

Classes 10 and 11 also included participants with higher SES, younger participants, and those who perceived their loneliness to be more controllable, compared to participants in classes 6 and 9, where duration was more long lasting. This suggests that having high SES provides people with resources that help them get out of loneliness—though the opposite cannot be ruled out: That is, having long lasting loneliness can have a negative impact on one’s perceived social status. These findings also suggest that young people are better able to terminate loneliness experiences, even if those are frequent and intense. 

The finding regarding controllability indicates that people who feel loneliness is under their control (either because it really is or because they are predisposed to believe it to be so) are better equipped to address it quickly. It may also suggest that being able to address loneliness quickly nurtures the belief that it is under one’s control, something that has been highlighted before [25]. In sum, the duration of loneliness is associated with differences in social capital, relational mobility, health, perceived controllability of loneliness, gender, age, and social status.

Importantly, we showed that different subtypes of loneliness experiences could be identified based on the different aspects of severity of loneliness. Duration of loneliness seemed to be particularly important in distinguishing subtypes, with variation between groups often defined in terms of their loneliness lasting several days (class 10), months (class 11), or many years (classes 6 and 9). Indeed, in the current study we found that ratings of loneliness based on frequency, intensity, and duration were highly correlated with each other. Indeed, in the LCA, scores on frequency and intensity were always in the same direction, with scores for how intensive the experience loneliness is corresponding with reports of how frequently the negative feeling or behaviour occurred. That suggests, while those different aspects of severity are important for understanding other internalising experiences such as depression and anxiety, they may not be so distinctive for understanding loneliness. That said, we have already pointed out that a defined timeframe is not provided for participants for evaluating the frequency of loneliness for most loneliness measures, and that may have meant that, here, participants found it difficult to distinguish between the terms of frequency and intensity. Scale development experts have highlighted this issue for depression [26] and suggested that mental representations of frequency and intensity are imprecise. Thus, future work should (1) explore how ratings of loneliness in terms of frequency and intensity are related once a defined time slot for frequency is provided for participants, and (2) examine how response categories should work to provide more precision in the reporting of loneliness. 

Duration seemed to be particularly important for predicting loneliness profiles in the current sample, suggesting that there are individual differences in how loneliness persists over time. How duration of loneliness is prospectively related to mental and physical health, and well-being outcomes, will need to be explored in future work so that we get a clear picture of what constitutes loneliness that persists for longer than would be expected. Such work is important for helping decide who receives support to address loneliness and when. 

### 4.2. Lonely vs. Not Lonely

Our analyses also compared the profiles of ‘lonely’ (classes 6, 9, 10, and 11) and ‘non-lonely’ (the remaining) participants. The differences between the profiles referred to similar variables. Compared with ‘non-lonely’ participants, those who had high scores on at least one of the loneliness dimensions were more likely to spend time alone, have more experiences with discrimination, have worse health, lower social capital, and lower relational mobility, be in a relationship, be male, and be younger. The associations that have been examined before, with the exception of gender, are consistent with previous research (ONS, 2018). In addition, this comparison revealed that the life events of the previous year played a role when differentiating between feeling or not feeling lonely in that more challenging life events were associated with more loneliness. The measure of life events included a whole range of life changes, some of which have been the object of previous studies in relation to loneliness (e.g., motherhood [27,28]), others less so (e.g., changes at work, or change of residence). Future research would do well to dedicate more attention to the role that specific life changes might play in loneliness experiences, and whether the same event differentially impacts at different ages/life stages. 

Interestingly, participants who reported feeling loneliness of short duration (Classes 10 and 11) also differed from ‘non-lonely’ participants by perceiving loneliness as less controllable. It is likely that those who experienced loneliness frequently and intensely, but not for a long time, were aware that their frequent and intense experiences emerge from a variety of factors, some of which, at least, they do not control. By contrast, ‘non-lonely’ participants relied on the value of self-control that is dominant in a society such as the UK to indicate that loneliness was largely controllable. Finally, participants who reported high frequency and intensity of loneliness differed from ‘non-lonely’ participants by reporting that at least some of their experiences with loneliness were positive and led to positive outcomes. Indeed, people who have ample experiences with loneliness that they can reflect on are likely to have sufficient variety within these to sample both positive and negative events, whereas those who have no experience, or few events to recall, are more likely to rely on stereotypical associations, or the social stigma associated with loneliness, and see it as a negative experience [29,30].

### 4.3. Strengths and Limitations of the Current Study Design

Strengths of this study reside in the large sample size and the use of standardized self-report instruments. Interpretation of our findings is limited by the cross-sectional nature of the study, which does not permit causal conclusions. We have been mindful of that when making interpretations of our findings and have noted the need for prospective research to provide a clearer picture of what causes what over time. While our findings may be taken as an indication that our measure of loneliness severity are valid, the duration and intensity items were simply adaptations of the UCLA (frequency) items. Future studies should examine the psychometric properties of the different aspects of loneliness severity, particularly given recent work highlighting the need for measurement invariance testing of the original UCLA items [7].

Our sample was obtained through online recruitment, providing several advantages over traditional data collection methods, including greater sample diversity as well as efficiency, affordability, and ease in collecting large samples of data. However, online data collection can affect data integrity; reduced engagement with the researcher means the participant can provide dishonest responses and repeat responders provide an additional challenge [31]. In our study, we reduced the likelihood of those issues impacting survey results by not providing monetary incentives to take part in the study and by collecting IP addresses in line with GDPR to examine whether we had repeat responders. Other biases may be evident in the current data. As data were collected via a collaboration with the BBC, the sample was selected because participants had an interest in the topic and listened to the BBC. 

We restricted our analyses to participants who were living in the UK, which was important given cultural variation in the experience of loneliness within this sample [10]. That means, however, that our findings are restricted to adults in a specific country (UK) and future empirical work should explore the distinct dimensions of loneliness severity in other countries and among people younger than 16 years of age. 

### 4.4. Implications for Research and Practice

Our findings suggest that, moving forward, researchers should consider different aspects of loneliness severity. First, duration of loneliness appears to be particularly important. In the current study, participants responded to items on the four-item UCLA by noting how long that feeling lasted when it occurred; they noted 1 = hours, 2 = days, 3 = weeks, 4 = months, 5 = longer. Those response categories were chosen because they are common options on measures of depression and anxiety, and, while our analyses showed scores could differentiate participants into different profiles, further work is needed to determine whether the response categories are the correct ones. 

Second, in the present study we found that loneliness ratings based on intensity and frequency were highly correlated with each other, and, in our LCA, scores on frequency and intensity were always in the same direction. Given that finding, future work should explore whether these different aspects of loneliness have dissimilar predictive roles, or whether a composite index of severity based on the sum of frequency, duration, and intensity is a more suitable measure of loneliness severity. Considering the distinct profiles in the current study, we suggest, instead, that researchers examine how people interpret items about frequency and intensity of loneliness. There might be situations where intensity ratings are the preferred metric, and that may be particularly the case in applied intervention work. For example, where intervention effects are assessed daily or throughout the day it may not make sense to assess the frequency or duration of loneliness. 

## 5. Conclusions

The results of the current study support a clear recommendation for examining different aspects of the severity of loneliness. Future work should further explore the different aspects of loneliness, which will be particularly important for deciding when and for whom loneliness interventions are provided. Further research should also investigate to what extent our results generalize to different age groups and people from different cultures, and how the different aspects of severity of loneliness are distinct across groups.

## Figures and Tables

**Table 1:** Characteristics of final sample used in the current study.

Male %		29.5%
Age 16–34 years %		16.8%
Age 65+ years %		19.9%
Age range in years		16–99
Unemployed %		5.1%
Number of hours spent alone (in last 24 h)	Mean/SD	12.00 (7.33)
Single %		52.8%
Carer %		8.3%
Immigrant to the UK %		9.0%
Sexual Minority %		9.7%
Negative life events in the last year ^ø^		142.32 (98.75)
Is loneliness ever positive % yes or sometimes		41.5%
Is loneliness controllable ^‡^	Mean/SD	2.88 (0.993)
Is loneliness changeable ^‡^	Mean/SD	3.13 (1.02)
Everyday discrimination ^ø^	Mean/SD	2.46 (1.02)
Social Capital ^ø^	Mean/SD	3.09 (0.789)
Relational Mobility ^ø^	Mean/SD	3.92 (0.739)
Health ^§^	Mean/SD	3.26 (1.09)

**Notes**: ^ø^ Scored according to the original recommendations (life events: 15; discrimination: 16; social capital: 17; relational mobility: 18); ^‡^ Rated 1 (strongly disagree) to 5 (strongly agree); ^§^ Rated as follows: 1 = poor, 2 = fair, 3 = good, 4 = very good, or 5 = excellent.

**Table 2:** Sample means/SDs (by age) for the three different aspects of severity of loneliness.

Severity of Loneliness				Age			
	16–24 years	25–34 years	35–44 years	45–54 years	55–64 years	65–74 years	75+ years
Frequency	3.15 (1.01)	2.99 (1.04)	3.05 (1.08)	2.97 (1.14)	2.88 (1.16)	2.80 (1.19)	2.72 (1.17)
Intensity	3.49 (1.10)	3.34 (1.15)	3.27 (1.19)	3.15 (1.26)	3.01 (1.30)	2.85 (1.31)	2.66 (1.31)
Duration	2.56 (1.32)	2.50 (1.38)	2.60 (1.47)	2.52 (1.55)	2.36 (1.56)	2.19 (1.57)	1.99 (1.52)

**Table 3:** Correlations (by age) between the three different aspects of severity of loneliness.

	Severity of Loneliness	
Age Group	Intensity	Duration
**16–24 years**		
Frequency	0.655	0.709
Intensity		0.581
**25–34 years**		
Frequency	0.689	0.750
Intensity		0.628
**35–44 years**		
Frequency	0.744	0.776
Intensity		0.668
**45–54 years**		
Frequency	0.770	0.804
Intensity		0.705
**55–64 years**		
Frequency	0.810	0.816
Intensity		0.741
**65–74 years**		
Frequency	0.828	0.820
Intensity		0.755
**75+ years**		
Frequency	0.840	0.797
Intensity		0.748

**Notes**: all correlations significant at *p* < 0.001.

**Table 4:** Table of fit statistics and entropy for each class model.

K	ll	AIC	BIC	ssaBIC	LMR-LRT	Entropy
1	−156,569.21	313,150.42	313,201.18	313,182.12	n/a	n/a
2	−126,147.13	252,314.25	252,398.86	252,367.08	<0.0001	0.874
3	−111,618.37	223,264.73	223,383.18	223,338.69	<0.0001	**0.884**
4	−104,198.04	208,432.07	208,584.36	208,527.16	<0.0001	0.875
5	−101,139.27	202,322.55	202,508.68	202,438.77	<0.0001	0.855
6	−99,421.02	198,894.04	199,114.02	199,031.39	<0.0001	0.846
7	−97,783.24	195,626.48	195,880.30	195,784.96	<0.0001	0.837
8	−96,816.670	193,701.39	193,989.05	193,881.00	0.0001	0.827
9	−96,174.58	192,425.16	192,746.66	192,625.90	0.0001	0.816
10	−95,608.901	191,301.81	191,657.16	191,523.68	0.036	0.818
11	−95,121.83	190,335.66	190,724.85	190,578.66	**0.041**	0.811
12	−94,645.28	189,390.57	189,813.60	189,654.70	0.525	0.809
13	−94,145.11	188,398.22	188,855.10	188,683.49	0.160	0.811
14	**−93,680.99**	**187,477.99**	**187,968.71**	**187,784.38**	0.036	0.812

**Notes**. bolded numbers indicate better model fit; k = number of profiles specified in the model; ll = loglikelihood; AIC = Akaike information criteria; BIC = Bayesian information criteria; ssaBIC = sample size adjusted BIC; LMR-LRT = Lo-Mendell-Rubin adjusted likelihood ratio test.

**Table 5:** Means on different aspects of severity of loneliness for each class identified in the final LCA.

	Class 1	Class 2	Class 3	Class 4	Class 5	Class 6	Class 7	Class 8	Class 9	Class 10	Class 11
Number of participants	1645	2865	5956	5689	5730	552	3108	4086	1854	945	2484
Proportion of sample (%)	4.7	8.2	17.1	16.3	16.4	1.6	8.9	11.7	5.3	2.7	7.1
Frequency of Loneliness	2.98	3.54	1.17	2.28	1.75	3.85	3.48	2.88	4.74	4.28	4.17
Intensity of Loneliness	3.07	3.65	1.16	2.38	1.78	3.42	3.41	2.88	4.65	4.25	4.13
Duration of Loneliness	1.47	2.11	0.16	1.43	0.82	4.67	3.21	2.26	4.80	2.72	3.91

**Notes:** Highlighted columns are the classes scoring highest on the severity of loneliness measures.

**Table 6:** Odds ratios (and 95% confidence intervals) showing the relationship between the lonely classes and other classes for the predictor variables.

**Odds Ratio Estimate 95% CI Class 6 Comparison**											
	**Class 1**	**Class 2**	**Class 3**	**Class 4**	**Class 5**	**Class 6**	**Class 7**	**Class 8**	**Class 9**	**Class 10**	**Class 11**
**Covariates**											
SES	**1.13 (1.03–1.24)**	1.07 (0.98–1.17)	**1.34 (1.23–1.47)**	**1.16 (1.07–1.27)**	**1.22 (1.12–1.32)**		1.06 (0.97–1.16)	1.08 (0.99–1.17)	0.92 (0.84–1.00)	**1.09 (1.01–1.12)**	**1.15 (1.07–1.25)**
Dependents	0.90 (0.60–1.35)	0.96 (0.67–1.39)	0.78 (0.55–1.12)	0.90 (0.64–1.28)	0.78 (0.55–1.11)		1.08 (0.75–1.56)	0.90 (0.63–1.30)	1.23 (0.84–1.79)	0.95 (0.63–1.44)	1.13 (0.77–1.67)
Carer	**0.45 (0.25–0.83)**	0.66 (0.41–1.07)	0.71 (0.44–1.14)	0.71 (0.46–1.12)	**0.60 (0.38–0.95)**		0.91 (0.57–1.44)	0.71 (0.45–1.14)	0.71 (0.43–1.16)	0.68 (0.39–1.18)	0.69 (0.41–1.14)
Hours spent alone	**0.97 (0.94–1.00)**	**0.97 (0.94–1.00)**	**0.88 (0.85–0.90)**	**0.93 (0.91–0.96)**	**0.90 (0.87–0.93)**		0.98 (0.95–1.01)	**0.94 (0.92–0.97)**	**1.03 (1.00–1.06)**	**1.05 (1.00–1.07)**	**1.03 (1.00–1.05)**
Life events (Z-score)	1.13 (0.94–1.37)	**1.24 (1.04–1.48)**	**0.76 (0.63–0.91)**	1.03 (0.86–1.23)	0.89 (0.75–1.07)		1.17 (0.98–1.40)	1.04 (0.87–1.25)	**1.24 (1.03–1.49)**	**1.26 (1.04–1.53)**	**1.26 (1.04–1.52)**
Individual can change loneliness	1.14 (0.91–1.42)	1.06 (0.87–1.29)	**1.37 (1.12–1.68)**	**1.26 (1.04–1.53)**	**1.31 (1.08–1.60)**		1.10 (0.90–1.34)	1.14 (0.94–1.40)	0.91 (0.73–1.12)	1.01 (0.80–1.26)	0.99 (0.80–1.23)
Individual can control loneliness	0.93 (0.74–1.18)	1.05 (0.84–1.30)	**1.46 (1.18–1.80)**	1.07 (0.87–1.32)	1.20 (0.98–1.48)		0.98 (0.79–1.22)	1.05 (0.84–1.30)	0.98 (0.80–1.20)	**0.77 (0.61–0.99)**	**0.77 (0.57–1.03)**
Prejudice	**0.70 (0.59–0.83)**	0.98 (0.85–1.14)	**0.40 (0.34–0.47)**	**0.61 (0.53–0.70)**	**0.50 (0.43–0.58)**		0.92 (0.79–1.06)	**0.77 (0.66–0.89)**	**1.44 (1.24–1.68)**	**1.30 (1.11–1.53)**	**1.36 (1.10–1.55)**
Self-rated health	**1.29 (1.10–1.51)**	**1.22 (1.05–1.42)**	**1.73 (1.49–2.02)**	**1.45 (1.25–1.68)**	**1.55 (1.33–1.79)**		1.12 (0.96–1.31)	**1.32 (1.14–1.54)**	0.91 (0.78–1.07)	**1.15 (0.97–1.37)**	1.03 (0.88–1.21)
Social capital/trust	**1.27 (1.05–1.35)**	**1.27 (1.03–1.56)**	**1.96 (1.59–2.41)**	**1.48 (1.21–1.82)**	**1.74 (1.42–2.13)**		1.16 (0.94–1.43)	**1.43 (1.16–1.76)**	0.87 (0.69–1.09)	0.95 (0.75–1.21)	**1.27 (1.05–1.54)**
Relational Mobility	**1.47 (1.16–1.87)**	**1.32 (1.05–1.65)**	**2.35 (1.88–2.95)**	**1.49 (1.20–1.85)**	**1.60 (1.28–1.99)**		1.16 (0.92–1.45)	1.23 (0.99–1.54)	1.18 (0.93–1.50)	**1.34 (1.04–1.73)**	1.16 (0.90–1.48)
UK immigrant	0.94 (0.52–1.70)	0.99 (0.58–1.72)	1.01 (0.59–1.74)	1.08 (0.64–1.82)	0.97 (0.57–1.66)		1.13 (0.65–1.97)	1.08 (0.63–1.86)	1.23 (0.70–2.18)	1.08 (0.59–1.98)	0.82 (0.45–1.49)
Loneliness as positive	0.84 (0.60–1.18)	1.24 (0.91–1.70)	**0.42 (0.31–0.57)**	**0.68 (0.50–0.91)**	**0.52 (0.38–0.70)**		0.93 (0.68–1.27)	0.80 (0.59–1.09)	**1.68 (1.21–2.34)**	**1.27 (1.19–1.82)**	**1.59 (1.13–2.23)**
Employed	1.05 (0.72–1.53)	0.83 (0.59–1.17)	1.09 (0.77–1.54)	1.00 (0.72–1.40)	1.03 (0.74–1.44)		0.98 (0.69–1.40)	1.04 (0.74–1.46)	0.80 (0.56–1.13)	0.99 (0.67–1.45)	0.81 (0.56–1.16)
Male	**0.60 (0.42–0.84)**	**0.71 (0.51–0.96)**	**0.53 (0.39–0.72)**	**0.67 (0.50–0.90)**	**0.65 (0.48–0.88)**		**0.82 (0.60–1.13)**	**0.77 (0.57–1.06)**	**0.81 (0.59–1.11)**	**0.65 (0.45–0.92)**	**0.65 (0.46–0.91)**
Age 16–34	**2.64 (1.44–4.83)**	**2.84 (1.59–5.09)**	**3.57 (1.99–6.41)**	**2.29 (1.29–4.07)**	**2.60 (1.46–4.64)**		**2.00 (1.10–3.64)**	**2.10 (1.17–3.78)**	**1.56 (1.15–2.85)**	**2.41 (1.31–4.42)**	**1.86 (1.00–3.46)**
Age over 65	0.94 (0.59–1.50)	0.78 (0.51–1.20)	**1.70 (1.13–2.58)**	1.30 (0.87–1.93)	1.47 (0.99–2.21)		0.76 (0.49–1.18)	1.12 (0.74–1.71)	**0.67 (0.43–1.04)**	**0.56 (0.33–0.94)**	**0.64 (0.40–1.01)**
Sexual Minority	1.32 (0.74–2.37)	1.51 (0.88–2.61)	1.11 (0.63–1.96)	1.25 (0.73–2.13)	1.21 (0.70–2.07)		1.43 (0.82–2.49)	1.37 (0.79–2.37)	1.41 (0.81–2.47)	1.37 (0.76–2.48)	1.57 (0.89–2.77)
Not in a relationship	**1.81 (1.20–2.73)**	**1.67 (1.15–2.43)**	1.10 (0.76–1.60)	**1.58 (1.10–2.26)**	**1.66 (1.16–2.38)**		1.33 (0.91–1.94)	**1.45 (1.00–2.10)**	**1.52 (1.03–2.24)**	1.36 (0.89–2.09)	1.32 (0.89–1.95)
**Odds Ratio Estimate 95% CI Class 9 Comparison**											
	**class 1**	**class 2**	**class 3**	**class 4**	**class 5**	**class 6**	**class 7**	**class 8**	**class 9**	**class 10**	**class 11**
**Covariates**											
SES	**1.23 (1.15–1.31)**	**1.17 (1.11–1.23)**	**1.47 (1.38–1.56)**	**1.27 (1.21–1.33)**	**1.33 (1.26–1.40)**		**1.15 (1.10–1.22)**	**1.17 (1.11–1.24)**		**1.10 (1.03–1.18)**	**1.14 (1.08–1.20)**
Dependents	**0.73 (0.54–0.98)**	**0.78 (0.62–0.99)**	**0.63 (0.51–0.80)**	**0.73 (0.59–0.91)**	**0.63 (0.51–0.79)**		0.88 (0.70–1.10)	**0.73 (0.58–0.93)**		0.77 (0.57–1.05)	0.92 (0.72–1.18)
Carer	0.64 (0.39–1.06)	0.94 (0.67–1.31)	1.00 (0.71–1.41)	1.01 (0.74–1.37)	0.85 (0.61–1.17)		1.28 (0.94–1.75)	1.01 (0.72–1.42)		0.96 (0.62–1.48)	0.97 (0.69–1.37)
Hours spent alone	**0.94 (0.92–0.96)**	**0.94 (0.92–0.95)**	**0.85 (0.83–0.87)**	**0.90 (0.89–0.92)**	**0.87 (0.86–0.89)**		**0.95 (0.93–0.96)**	**0.92 (0.90–0.93)**		**0.98 (0.97–0.99)**	**0.97 (0.96–0.99)**
Life events (Z-score)	0.92 (0.82–1.03)	1.00 (0.92–1.10)	**0.61 (0.55–0.68)**	**0.83 (0.76–0.91)**	**0.72 (0.66–0.79)**		0.95 (0.87–1.04)	**0.84 (0.77–0.93)**		1.02 (0.91–1.14)	1.02 (0.93–1.13)
Individual can change loneliness	**1.26 (1.07–1.47)**	**1.17 (1.03–1.33)**	**1.52 (1.33–1.73)**	**1.39 (1.23–1.57)**	**1.45 (1.28–1.64)**		**1.21 (1.07–1.37)**	**1.26 (1.11–1.44)**		1.11 (0.94–1.31)	1.10 (0.96–1.26)
Individual can control loneliness	0.96 (0.82–1.13)	1.08 (0.95–1.23)	**1.50 (1.32–1.71)**	1.11 (0.98–1.25)	**1.24 (1.10–1.40)**		1.01 (0.87–1.16)	1.08 (0.95–1.23)		**0.72 (0.56–0.81)**	**0.73 (0.57–0.81)**
Prejudice	**0.48 (0.43–0.55)**	**0.68 (0.62–0.75)**	**0.28 (0.25–0.31)**	**0.42 (0.39–0.46)**	**0.35 (0.32–0.38)**		**0.64 (0.58–0.70)**	**0.53 (0.48–0.59)**		**0.80 (0.71–0.91)**	**0.80 (0.73–0.89)**
Self-rated health	**1.41 (1.26–1.58)**	**1.34 (1.22–1.47)**	**1.90 (1.72–2.10)**	**1.59 (1.45–1.73)**	**1.69 (1.55–1.86)**		**1.23 (1.12–1.35)**	**1.45 (1.31–1.60)**		**1.26 (1.12–1.43)**	**1.13 (1.02–1.25)**
Social capital/trust	**1.23 (1.04–1.46)**	**1.46 (1.27–1.68)**	**2.25 (1.95–2.60)**	**1.71 (1.50–1.95)**	**2.00 (1.75–2.28)**		**1.33 (1.16–1.53)**	**1.64 (1.42–1.89)**		1.10 (0.91–1.31)	**1.23 (1.01–1.43)**
Relational Mobility	**1.25 (1.06–1.48)**	1.12 (0.96–1.29)	**2.00 (1.71–2.33)**	**1.26 (1.10–1.45)**	**1.35 (1.17–1.56)**		0.98 (0.85–1.13)	1.05 (0.90–1.21)		**1.24 (1.04–1.48)**	0.98 (0.83–1.16)
UK immigrant	0.76 (0.52–1.13)	0.81 (0.59–1.11)	0.82 (0.60–1.13)	0.87 (0.65–1.17)	0.79 (0.58–1.07)		0.92 (0.67–1.26)	0.88 (0.64–1.22)		0.88 (0.59–1.31)	**0.67 (0.46–0.96)**
Loneliness as positive	**0.50 (0.39–0.64)**	**0.74 (0.60–0.91)**	**0.25 (0.20–0.31)**	**0.40 (0.33–0.49)**	**0.31 (0.25–0.37)**		**0.55 (0.45–0.68)**	**0.48 (0.39–0.59)**		0.76 (0.58–1.00)	**0.95 (0.65–1.00)**
Employed	**1.32 (1.01–1.73)**	1.04 (0.84–1.30)	**1.37 (1.09–1.72)**	**1.26 (1.01–1.55)**	**1.30 (1.05–1.60)**		1.24 (0.99–1.54)	**1.31 (1.04–1.64)**		1.24 (0.94–1.64)	1.01 (0.80–1.28)
Male	**0.74 (0.58–0.95)**	0.87 (0.71–1.07)	**0.65 (0.53–0.81)**	**0.83 (0.69–1.00)**	**0.81 (0.67–0.97)**		1.02 (0.84–1.24)	0.96 (0.78–1.18)		**0.80 (0.61–0.98)**	**0.80 (0.65–1.00)**
Age 16–34	**1.70 (1.24–2.31)**	**1.83 (1.41–2.36)**	**2.29 (1.75–3.01)**	**1.47 (1.14–1.89)**	**1.67 (1.29–2.16)**		1.29 (0.98–1.69)	**1.35 (1.01–1.78)**		**1.55 (1.12–2.14)**	**1.20 (1.09–1.41)**
Age over 65	1.40 (0.98–2.00)	1.16 (0.86–1.56)	**2.54 (1.89–3.40)**	**1.93 (1.47–2.52)**	**2.19 (1.67–2.89)**		1.13 (0.84–1.53)	**1.67 (1.24–2.25)**		0.83 (0.55–1.26)	0.96 (0.70–1.31)
Sexual Minority	0.98 (0.66–1.33)	1.07 (0.81–1.42)	0.80 (0.57–1.10)	0.88 (0.67–1.16)	0.86 (0.64–1.14)		1.01 (0.76–1.34)	0.97 (0.71–1.31)		0.97 (0.68–1.40)	1.11 (0.83–1.49)
Not in a relationship	1.19 (0.89–1.60)	1.10 (0.87–1.39)	**0.73 (0.57–0.93)**	1.04 (0.83–1.29)	1.09 (0.87–1.37)		0.88 (0.70–1.11)	0.95 (0.75–1.21)		0.90 (0.65–1.23)	0.87 (0.67–1.11)
**Odds Ratio Estimate 95% CI Class 10 Comparison**											
	**class 1**	**class 2**	**class 3**	**class 4**	**class 5**	**class 6**	**class 7**	**class 8**	**class 9**	**class 10**	**class 11**
**Covariates**											
SES	**1.11 (1.03–1.20)**	1.06 (0.99–1.14)	**1.33 (1.24–1.43)**	**1.15 (1.08–1.23)**	**1.20 (1.12–1.28)**		1.05 (0.98–0.99)	1.06 (0.99–1.14)			1.04 (0.98–1.11)
Dependents	0.94 (0.22–1.34)	1.01 (0.74–1.39)	0.82 (0.61–1.10)	0.95 (0.71–1.26)	0.82 (0.61–1.09)		1.14 (0.84–1.54)	0.95 (0.70–1.28)			1.19 (0.86–1.64)
Carer	0.67 (0.38–1.18)	0.98 (0.62–1.55)	1.05 (0.67–1.62)	1.05 (0.70–1.59)	0.88 (0.58–1.35)		1.33 (0.87–2.04)	1.05 (0.68–1.63)			1.01 (1.64–1.60)
Hours spent alone	**0.95 (0.93–0.98)**	**0.95 (0.93–0.98)**	**0.86 (0.84–0.89)**	**0.92 (0.90–0.94)**	**0.89 (0.87–0.91)**		**0.96 (0.94–0.98)**	**0.93 (0.91–0.95)**			1.00 (0.97–1.02)
Life events (Z-score)	0.90 (0.79–1.02)	0.98 (0.88–1.10)	**0.60 (0.53–0.68)**	**0.82 (0.73–0.91)**	**0.71 (0.63–0.79)**		0.93 (0.83–1.04)	**0.83 (0.74–0.93)**			1.00 (0.89–1.13)
Individual can change loneliness	1.13 (0.94–1.36)	1.05 (0.89–1.24)	**1.37 (1.16- 1.60)**	**1.25 (1.08–1.46)**	**1.31 (1.12–1.52)**		1.09 (0.93–1.28)	1.14 (0.97–1.33)			0.99 (0.83–1.17)
Individual can control loneliness	1.18 (0.97–1.45)	**1.33 (1.10–1.60)**	**1.84 (1.54–2.20)**	**1.36 (1.15–1.61)**	**1.52 (1.28–1.81)**		**1.25 (1.04–1.49)**	**1.32 (1.11–1.58)**			**1.23 (1.01–1.50)**
Prejudice	**0.54 (0.47–0.61)**	**0.76 (0.68–0.84)**	**0.31 (0.27–0.34)**	**0.47 (0.42–0.52)**	**0.39 (0.35–0.428)**		**0.70 (0.63–0.78)**	**0.59 (0.53–0.66)**			**0.89 (0.79–0.99)**
Self-rated health	1.12 (0.98–1.27)	1.06 (0.94–1.20)	**1.51 (1.36–1.70)**	**1.26 (1.13–1.40)**	**1.34 (1.20–1.50)**		0.97 (0.87–1.10)	**1.15 (1.02–1.29)**			**0.79 (0.69–0.92)**
Social capital/trust	1.12 (0.92–1.37)	**1.33 (1.11–1.59)**	**2.06 (1.73–2.45)**	**1.56 (1.32–1.84)**	**1.82 (1.55–2.15)**		**1.22 (1.02–1.45)**	**1.50 (1.26–1.78)**			**1.22 (1.03–1.45)**
Relational Mobility	1.10 (0.91–1.33)	0.98 (0.81–1.18)	**1.75 (1.46–2.10)**	1.11 (0.94–1.31)	**1.19 (1.00–1.41)**		0.86 (0.72–1.03)	0.92 (0.77–1.09)			**0.76 (0.61–0.95)**
UK immigrant	0.88 (0.55–1.36)	0.92 (0.61–1.38)	0.93 (0.63–1.38)	0.99 (0.63–1.38)	0.90 (0.62–1.31)		1.05 (0.70–1.56)	1.00 (0.67–1.48)			0.76 (0.48–1.19)
Loneliness as positive	**0.66 (0.50–0.88)**	0.98 (0.74–1.29)	**0.33 (0.25–0.42)**	**0.53 (0.41–0.68)**	**0.41 (0.32–0.52)**		**0.73 (0.56–0.95)**	**0.63 (0.48–0.81)**			1.25 (0.93–1.66)
Employed	1.06 (0.78–1.45)	0.84 (0.63–1.18)	1.10 (0.84–1.45)	1.02 (0.78–1.32)	1.04 (0.80–1.36)		0.99 (0.75–1.32)	1.05 (0.80–1.38)			0.82 (0.61–1.09)
Male	0.93 (0.69–1.25)	1.09 (0.83–1.44)	0.82 (0.63–1.07)	1.04 (0.81–1.33)	1.01 (0.78–1.30)		1.28 (0.98–1.67)	1.20 (0.92–1.56)			1.01 (0.76–1.34)
Age 16–34	1.10 (0.78–1.54)	1.18 (0.87–1.61)	**1.48 (1.09–2.01)**	0.95 (0.71–1.27)	1.08 (0.81–1.45)		0.83 (0.61–1.14)	0.87 (0.64–1.19)			0.78 (0.55–1.09)
Age over 65	1.68 (1.06–2.65)	1.39 (0.90–2.15)	**3.04 (2.02–4.58)**	**2.31 (1.56–3.43)**	**2.63 (1.77–3.91)**		1.36 (0.89–2.08)	**2.01 (1.33–3.02)**			1.15 (0.74–1.79)
Sexual Minority	0.97 (0.64–1.46)	1.10 (0.76–1.60)	0.81 (0.55–1.21)	0.91 (0.64–1.29)	0.88 (0.62–1.26)		1.04 (0.72–1.51)	1.00 (0.69–1.44)			1.15 (0.79–1.67)
Not in a relationship	1.33 (0.94–1.89)	1.23 (0.90–1.69)	0.81 (0.60–1.10)	1.16 (0.86–1.55)	1.22 (0.91–1.64)		0.98 (0.72–1.34)	1.06 (0.78–1.45)			0.97 (0.69–1.34)
**Odds Ratio Estimate 95% CI Class 11 Comparison**											
	**class 1**	**class 2**	**class 3**	**class 4**	**class 5**	**class 6**	**class 7**	**class 8**	**class 9**	**class 10**	**class 11**
**Covariates**											
SES	**1.18 (1.11–1.25)**	**1.12 (1.07–1.18)**	**1.41 (1.33–1.49)**	**1.22 (1.16–1.28)**	**1.27 (1.21–1.24)**		**1.11 (1.05–1.17)**	**1.13 (1.07–1.19)**			
Dependents	0.79 (0.59–1.06)	0.85 (0.68–1.06)	**0.69 (0.55–0.86)**	**0.80 (0.65–0.98)**	**0.69 (0.56–0.85)**		0.95 (0.76–1.20)	**0.80 (0.64–1.00)**			
Carer	0.66 (0.40–1.09)	0.96 (0.70–1.34)	1.03 (0.74–1.43)	1.04 (0.76–1.39)	0.87 (0.64–1.19)		1.32 (0.96–1.81)	1.04 (0.75–1.44)			
Hours spent alone	**0.95 (0.93–0.97)**	**0.95 (0.94–0.97)**	**0.87 (0.85–0.89)**	**0.92 (0.91–0.93)**	**0.89 (0.87–0.90)**		**0.96 (0.95–0.98)**	**0.93 (0.92–0.95)**			
Life events (Z-score)	**0.90 (0.81–1.00)**	0.98 (0.90–1.07)	**0.60 (0.54–0.66)**	**0.82 (0.75–0.89)**	**0.71 (0.65–0.77)**		0.93 (0.85–1.01)	**0.83 (0.76–0.90)**			
Individual can change loneliness	1.15 (0.99–1.33)	1.07 (0.95–1.07)	**1.38 (1.23–1.56)**	**1.27 (1.14–1.42)**	**1.32 (1.18–1.48)**		1.11 (0.98–1.25)	**1.15 (1.02–1.30)**			
Individual can control loneliness	1.09 (0.92–1.29)	**1.22 (1.06–1.40)**	**1.69 (1.48–1.94)**	**1.25 (1.10–1.42)**	**1.40 (1.23–1.60)**		1.14 (1.00–1.31)	**1.22 (1.06–1.40)**			
Prejudice	**0.60 (0.54–0.68)**	**0.85 (0.78–0.93)**	**0.34 (0.31–0.38)**	**0.53 (0.48–0.57)**	**0.43 (0.40–0.48)**		**0.79 (0.72–0.87)**	**0.66 (0.61–0.73)**			
Self-rated health	**1.25 (1.13–1.39)**	**1.19 (1.09–1.30)**	**1.68 (1.53–1.85)**	**1.41 (1.30–1.53)**	**1.50 (1.38–1.64)**		1.09 (0.99–1.20)	**1.28 (1.17–1.41)**			
Social capital/trust	1.00 (0.85–1.18)	**1.19 (1.05–1.35)**	**1.84 (1.62–2.09)**	**1.39 (1.24–1.57)**	**1.63 (1.45–1.84)**		1.08 (0.95–1.24)	**1.34 (1.18–1.52)**			
Relational Mobility	**1.28 (1.09–1.49)**	1.14 (1.00–1.30)	**2.04 (1.78–2.34)**	**1.29 (1.14–1.46)**	**1.38 (1.22–1.57)**		1.00 (0.87–1.15)	1.07 (0.94–1.22)			
UK immigrant	1.15 (0.76–1.72)	1.21 (0.87–1.69)	1.23 0.88–1.72)	1.31 (0.97–1.78)	1.19 (0.86–1.63)		1.38 (0.98–1.95)	1.32 (0.94–1.85)			
Loneliness as positive	**0.53 (0.42–0.67)**	**0.78 (0.64–0.96)**	**0.26 (0.22–0.32)**	**0.43 (0.36–0.51)**	**0.33 (0.27–0.39)**		**0.58 (0.48–0.72)**	**0.50 (0.41–0.61)**			
Employed	**1.30 (1.01–1.69)**	1.03 (0.83–1.27)	**1.35 (1.09–1.67)**	**1.25 (1.02–1.51)**	**1.28 (1.05–1.56)**		1.22 (0.97–1.52)	**1.29 (1.04–1.60)**			
Male	0.92 (0.72–1.18)	1.09 (0.89–1.33)	0.81 (0.66–1.00)	1.03 (0.86–1.24)	1.00 (0.83–1.21)		**1.27 (1.03–1.56)**	1.19 (0.97–1.46)			
Age 16–34	**1.42 (1.05–1.91)**	**1.53 (1.20–1.33)**	**1.91 (1.48–2.47)**	1.23 (0.97–1.55)	**1.40 (1.10–1.77)**		1.07 (0.82–1.41)	1.13 (0.87–1.47)			
Age over 65	**1.46 (1.02–2.09)**	1.21 (0.90–1.63)	**2.65 (1.98–3.55)**	**2.02 (1.55–2.63)**	**2.30 (1.75–3.02)**		1.19 (0.87–1.62)	**1.75 (1.30–2.35)**			
Sexual Minority	0.84 (0.60–1.18)	0.96 (0.74–1.25)	**0.71 (0.52–0.97)**	0.79 (0.62–1.03)	0.77 (0.59–1.00)		0.91 (0.68–1.21)	0.87 (0.65–1.16)			
Not in a relationship	**1.38 (1.03–1.84)**	**1.27 (1.02–1.59)**	0.84 (0.67–1.06)	1.20 (0.98–1.48)	**1.26 (1.02–1.56)**		1.01 (0.80–1.28)	1.10 (0.88–1.39)			

**Notes**: Bold = significant effect.

**Table 7:** Easy View: How are the lonely and non-lonely classes different to each other? What distinguishes one lonely class from the others?

Covariates	Loneliness Class Differences	Lonely Groups Compared to Non-Lonely Groups §
SES	10, 11 > 6, 9	6, 9, 10, 11 = lower SES
Dependents	NS	9, 11 = more dependents
Carer	NS	NS
Hours spent alone	9 > 10, 11 > 6	6, 9, 10, 11 = more time alone
Number of life events †	9, 10, 11 > 6	9, 10, 11 = more life events
Individual can change loneliness	NS	9 = less likely to think individuals can change their loneliness
Individual can control loneliness	6, 9 > 10 > 11	10, 11 = less likely to think individuals can control their loneliness
Experienced discrimination	9 > 10 > 11 > 6	6, 9, 10, 11 = experience more prejudice
Self-rated health	10 > 11 > 6, 9	6, 9, 10, 11 = worse health
Social capital/trust	11 > 6, 9, 10	6, 9, 10, 11 = less social capital
Relational Mobility	10 > 6, 9, 11	6, 9, 10, 11 = less relational mobility
UK immigrant	9 > 11	NS
Loneliness as positive	9 > 10, 11 > 6	9, 10, 11 = more likely to think loneliness could be positive
Employed	NS	9, 11 = more likely to be unemployed
Male	6 > 9 > 10, 11	6, 9 = more likely to be male
Age 16–34	10, 11 > 9 > 6	6, 9, = less likely to be aged 16–34 years
Age over 6	6 > 9 > 10, 11	9, 10, 11 = less likely to be over 65 years of age
Sexual Minority	NS	NS
Not in a relationship	6 > 9	6 = less likely to be in a relationship

**Notes**: § = most consistent patterns (more than half of the non-lonely groups); † = z score used in model; Class 6 = average frequency, average intensity, and long duration of loneliness; Class 9 = high frequency, high intensity, and long duration; Class 10 = high frequency, high intensity, and short duration; Class 11 = high frequency, high intensity, and average duration.

## Data Availability

These data are available upon request from the corresponding author.
